# Correlations of Cancer-Related Fatigue with Clinicopathological Features and Quality of Life in Gastric Cancer

**DOI:** 10.1155/2024/4850745

**Published:** 2024-05-14

**Authors:** Dong Liu, A-Dong Xia, Yue-Long Xing, Kai Zhang, Dan Chen

**Affiliations:** Department of Hepatobiliary and Pancreatic Gastroenterology, Affiliated Jinhua Hospital of Wenzhou Medical University, Jinhua People's Hospital, Jinhua, Zhejiang Province, China

## Abstract

**Objective:**

To explore the correlations of cancer-related fatigue (CRF) with clinicopathological features and quality of life in gastric cancer.

**Methods:**

Using a convenient sampling method, 230 patients with gastric cancer admitted to our hospital from March 2020 to July 2022 were collected. They were divided into the fatigue group (*n* = 152) and the nonfatigue group (*n* = 78) according to the presence/absence of CRF. Relevant data were collected and compared.

**Results:**

Statistically significant differences were found between the two groups in age ratio (*χ*^2^ = 41.671, *P* < 0.001), T stage ratio (*χ*^2^ = 9.973, *P* = 0.019), N stage ratio (*P* < 0.001), PS score (*P* < 0.001), and the degree of gastric cancer thickening (14.21 ± 3.32 vs. 12.12 ± 3.81 mm, *t* = 4.572, *P* < 0.001). Patients with gastric cancer had the lowest CRF Brief Fatigue Inventory (BFI) score for general activities (2.26 ± 0.37) and high scores for work activities (6.23 ± 0.24) and enjoyment of life (7.11 ± 1.34). Pearson's correlation analysis revealed a positive correlation between patient emotions and the CRF BFI score (*r* = 0.443, *P* = 0.001). Patients with mild, moderate, and severe CRF showed statistically significant differences in physical functioning (83.34 ± 21.12 vs. 65.23 ± 21.14 vs. 32.25 ± 17.29, *F* = 15.382, *P* < 0.001), role emotional (72.53 ± 21.21 vs. 67.33 ± 27.56 vs. 54.37 ± 26.45, *F* = 14.483, *P* < 0.001), fatigue (49.12 ± 18.44 vs. 54.61 ± 26.64 vs. 67.51 ± 14.27, *F* = 13.581, *P* < 0.001), bodily pain (56.56 ± 25.12 vs. 76.43 ± 21.71 vs. 80.32 ± 12.39, *F* = 14.582, *P* < 0.001), appetite reduction (57.45 ± 25.47 vs. 69.51 ± 16.21 vs. 76.23 ± 27.58, *F* = 14.592, *P* < 0.001), and overall health status and quality of life (67.21 ± 19.45 vs. 53.43 ± 22.32 vs. 43.43 ± 12.52, *F* = 16.494, *P* < 0.001). After chemotherapy, the average CRF BFI scores of the partial remission (PR), disease stability (SD), and disease progression (PD) groups all reduced than those before chemotherapy (all *P* < 0.05). At 3 months of follow-up, a comparison of the average CRF BFI scores with those before chemotherapy revealed a decrease in the SD and PR groups and an increase in the PD group.

**Conclusion:**

In conclusion, CRF is correlated with age, T stage, and N stage in gastric cancer. The later the T and N stages, the more significant the effect on fatigue. Moreover, CRF can also affect the quality of life in gastric cancer, and the severer the CRF, the poorer the quality of life.

## 1. Introduction

Globally, gastric cancer is one of the most common cancers, with over 1 million new cases annually [1]. In 2020, there were approximately 1.09 million new cases and 770,000 deaths related to gastric cancer worldwide [2]. The incidence of gastric cancer is notably high in China, exhibiting an increasing trend in recent years [3]. Most patients with gastric cancer do not exhibit obvious symptoms in the early stage. As the condition progresses, patients may experience symptoms such as pain, loss of appetite, and fatigue [4]. Gastric cancer is relatively sensitive to chemotherapy, and chemotherapeutics increase the 5-year survival rate of patients by 20% [5]. Neoadjuvant chemotherapy may provide patients with an opportunity for radical resection and long-term survival. However, while eliminating cancerous tissues, chemoradiotherapy can also cause huge damage to normal tissues and functions of the body. All patients experience varying degrees of side effects, such as nausea, vomiting, hearing loss, radiation-induced mucositis, diarrhea, and fatigue [6, 7], which increase the physical and psychological burden and seriously affect the quality of life of patients. Cancer-related fatigue (CRF) is unavoidable and one of the most painful symptoms for patients with cancer during and after treatment, with an incidence rate ranging from 7% to 75% [8, 9].

The National Comprehensive Cancer Center Network defines CRF as a distressing, persistent, subjective sense of physical, emotional, and/or cognitive tiredness or exhaustion related to cancer or cancer treatment that is not proportional to recent activity and interferes with usual functioning [10]. Cancer-related fatigue is different from general fatigue, as it cannot be counteracted by rest, and patients may even become more tired after rest [11]. At present, there are no effective drugs for this symptom, and it is a symptom that clinicians often overlook [12]. The pathological and physiological mechanisms underlying CRF remain unclear. However, research has demonstrated that it may be correlated with some common potential biological changes, and these physiological findings may even persist for several years after cancer treatment. The mechanistic hypotheses currently recognised include proinflammatory factors [13], hypothalamic–pituitary–adrenal axis dysregulation [14], circadian rhythm disorders [15, 16], skeletal muscle atrophy [17], and genetic factors [18].

Therefore, in the absence of a definite mechanism and effective treatment methods, the prevention and symptom improvement of CRF are extremely important. To the best of our knowledge, there are no articles reporting the correlation between CRF and quality of life in patients with gastric cancer. Thus, the present study investigates the CRF of patients with gastric cancer and analyzes its correlations with clinicopathological data and quality of life to provide a reference for improving CRF in patients with gastric cancer.

## 2. Subjects and Methods

### 2.1. Subjects

This is a single-center retrospective study. A convenience sampling method was used to select 230 patients with gastric cancer admitted to our hospital between March 2020 and July 2022 to participate in the study. The participants were divided into a fatigue group (*n* = 152) and a nonfatigue group (*n* = 78) according to the presence or absence of CRF. The inclusion criteria were as follows: (1) gastric cancer confirmed by pathological diagnosis [19]; (2) a Karnofksy performance status (KPS) score ≥ 60 or an Eastern Cooperative Oncology Group (ECOG) performance status score ≤ 2; (3) an age of 18-75 years; (4) an ability to complete scale investigation independently or with the assistance of others; (5) good compliance and an ability to cooperate with treatment and various examinations. The exclusion criteria included (1) mental disorders, restlessness, or dementia; (2) communication disorders; (3) severe memory loss; (4) missing values > 5% or regular answers in recovered questionnaires; (5) complications with other malignant tumors; and (6) suffering from severe underlying diseases of the heart, brain, and kidney. This study was approved by the ethics committee of our hospital, and all the included patients signed the informed consent.

### 2.2. Research Methods

#### 2.2.1. TNM Staging of Gastric Cancer Based on CT Imaging

TNM staging of the patients with gastric cancer was conducted based on CT imaging.

*T staging:* according to the 8th Edition AJCC Cancer Staging Manual and recommendations from domestic CSCO guidelines [20], stage T1 was determined by a visibly continuous and intact lowly enhanced band between the inner layer of highly enhanced tumor and the outer layer of slightly highly enhanced muscle (auxiliary sign: highly enhanced tumor did not exceed 50% of the full thickness of the gastric wall). Stage T2 was determined by the interruption and disappearance of the lowly enhanced band in the middle layer and slightly high enhancement of residual muscle in the outer layer (auxiliary sign: highly enhanced tumor exceeded 50% of the full thickness of the gastric wall). Stage T3 was determined by invasion into subserosal adipose tissue with a smooth serosal surface. Stage T4 was determined by invasion outside the serosa, with dense burrs, strip-like infiltration, and irregular nodules on the serosal surface. Based on the actual condition, the patients were only divided into T3 and T4 stages in this study.

*N staging:* according to the 8th Edition AJCC Cancer Staging Manual, N staging was carried out based on the short diameter of perigastric lymph nodes > 6 mm, and the short diameter of peripheral lymph nodes of the stomach > 8 mm, as well as auxiliary signs including a quasicircular shape, a significant enhancement, and a cluster of three or more lymph nodes. The size, number, and zoning of perigastric lymph nodes were recorded. Referring to the manual, N staging was performed based on the number of perigastric metastatic lymph nodes: NX: unevaluable regional lymph nodes; N0: no metastasis of regional lymph nodes; N1: metastatic lymph nodes in 1-2 regions; N2: metastatic lymph nodes in 3-6 regions; and N3: metastatic lymph nodes in 7 or more regions.

*M staging:* it is based on nonregional lymph node metastasis. According to the 8th Edition AJCC Cancer Staging Manual, with intra-abdominal nonregional lymph nodes, such as those involving the posterior region of the pancreatic head, pancreaticoduodenal, peripancreas, superior mesenteric artery, middle colic artery, para-abdominal aorta, or retroperitoneum, the disease was classified as stage M1. In the case of enlarged mediastinal lymph nodes, primary chest diseases were excluded. Metastases were considered when the above lymph node lesions showed an increase in size, enhancement, and quantity by comprehensive assessment in a follow-up physical examination.

*Peritoneal metastasis:* the appearance of peritoneal nodules, thickened and blurred peritoneum, and pelvic and abdominal masses, as well as indirect signs such as ascites, intestinal wall thickening, peritoneal convergence, and stripe-shaped shadows, was diagnosed as stage M1 after detection of condition deterioration in follow-up or confirmation by pathological biopsy with other primary lesions excluded.

#### 2.2.2. Classic Borrmann Classification and Criteria for Gastric Cancer

*Borrmann type I (fungating nodular type):* the tumor appears as a nodule or polyp, mainly growing into the cavity, with clear section boundaries, and enhancing depth limited to the mucosal layer.

*Borrmann type II (local ulcer type):* the ulcers were deep-seated, with a damlike protrusion at the edge. The tumor was localized, with clear boundaries, no obvious surrounding infiltration, and enhancing depth limited to the mucosal and submucosal layers.

*Borrmann type III (infiltrating ulcer type):* the ulcer bases were large, with unclear edges, obvious surroundings, and deep infiltration. The angle between the lesion edge and the gastric wall was often obtuse, and the enhancing depth exceeded the submucosal layer.

*Borrmann type IV (diffusely infiltrative type):* cancerous tissue diffusely infiltrated and grew in the gastric wall, with thickening and hardening of the infiltrated gastric wall, disappearance of folds, and flattening of the mucosa, sometimes accompanied by shallow ulcers. It forms a so-called linitis plastica when involving the entire stomach. The Borrmann classification of each patient on imaging was recorded. They were divided into proximal gastric cancer group and distal gastric cancer group based on the location of the lesion, and their composition ratios were compared.

#### 2.2.3. Measurement of Gastric Cancer Thickness

A length measurement tool from the PASC workstation was adopted. After adjusting the window width and level and magnifying locally, an enhanced cross-sectional image was taken, and three equidistant lines were used to divide the lesion into four equal segments at the axial position in the arterial phase during enhanced scanning centered on the lesion, with the midline passing through the center of the lesion. Measurements were conducted on each line three times, perpendicular to the gastric wall. The thickness of the lesion sites was measured, and the averages were taken and recorded.

#### 2.2.4. Determination of Lymph Node Metastasis

Lymph node metastasis was determined using pN staging: pN0 (cancer cells were not detected in lymph nodes), pN1 (cancer cells were detected in 1-2 lymph nodes), pN2 (cancer cells were detected in 3-6 lymph nodes), pN3a (cancer cells were detected in 7-15 lymph nodes), and pN3b (cancer cells were detected in ≥16 lymph nodes).

### 2.3. Data Collection

General data, pathological data, and quality of life data of the patients were collected. The general data consisted of gender, age, educational level, marital status, place of residence, employment, per capita monthly income of households, etc. The pathological data included degree of differentiation, TNM staging, Borrmann classification, degree of gastric cancer thickening, physical state (PS) score, efficacy of primary lesion, 6-cycle efficacy of lymph nodes, etc.

CRF was assessed using the Brief Fatigue Inventory (CRF BFI) [21], with a total of 9 items and a score ranging from 0 (not affected) to 10 (completely affected): 1-3 indicated mild CRF, 4-6 indicated moderate CRF, and 7-10 indicated severe CRF. The degree of fatigue was determined by the average score of the 9 items. The patients were divided into the CRF group (score > 0) and the non-CRF group (score = 0).

The assessment was conducted with the assistance of the patients, their family members, and medical staff a total of 4 times: 1 before the initial chemotherapy, 1 after 6 cycles of chemotherapy, 1 after 24 cycles of chemotherapy, and 1 during follow-up 3 months after chemotherapy.

The quality of life was measured by the European Organization for Research and Treatment of Cancer (EORTC) Quality of Life Questionnaire Core (QLQ-C30, V3.0), with a total of 30 items. Overall health status was scored by 2 items with a total of 7 options: a score of 1 for very poor and a score of 7 for very good. The higher the score, the better the quality of life. As for the rest of the items, there were a total of 4 options, with no problem scoring as 1, slightly serious problem as 2, serious problem as 3, and very serious problem as 4. The higher the score, the more serious the problem. To facilitate the comparison of scores in various fields, a range standardization method [22] was adopted for linear transformation.

After 6 cycles of chemotherapy, short-term efficacy was evaluated according to the Response Evaluation Criteria in Solid Tumors (RECIST, version 1.1) [23] and divided into complete remission (CR), partial remission (PR), disease stability (SD), and disease progression (PD).

### 2.4. Statistical Analysis

Statistical processing was carried out using SPSS 26.0. The K-S method was adopted to test normality. The measurement data satisfying the normal distribution were expressed as *x* ± *s*. Their comparisons between two groups were performed by the *t* test and among multiple groups by the single-factor analysis of variance. The enumeration data were expressed as frequency (*n*) or rate (%). Those satisfying the normal distribution were analyzed with the *χ*^2^ test, and those not satisfying the normal distribution were analyzed with Fisher's exact probability test. The ranked data were analyzed using the rank-sum test. Correlations were analyzed by Pearson correlation analysis. A two-tailed *P* < 0.05 was considered statistically significant.

## 3. Results

### 3.1. Comparison of General Data

In the CRF group (*n* = 152), there were 75 males and 77 females. The non-CRF group (*n* = 78) included 44 males and 34 females. A statistically significant difference was found in the age ratio between the two groups (*χ*^2^ = 41.671, *P* < 0.001). The two groups showed no statistically significant differences in gender, educational level, or marital status (*P* > 0.05), as listed in Table 1.

### 3.2. Comparison of Pathological Data

T stage ratio (*χ*^2^ = 9.973, *P* = 0.019), N stage ratio (*P* < 0.001), PS score (*P* < 0.001), and the degree of gastric cancer thickening (14.21 ± 3.32 vs. 12.12 ± 3.81 mm, *t* = 4.572, *P* < 0.001) showed statistically significant differences between the two groups. However, no statistically significant differences were observed in the degree of differentiation, lymph node metastasis, efficacy of primary lesion, or efficacy of lymph nodes (*P* > 0.05), as seen in Table 2.

### 3.3. Correlation between CRF BFI Score and Activities of Daily Living (ADLs)

Patients with gastric cancer had the lowest CRF BFI score for general activities (2.26 ± 0.37) and high scores for work activities (6.23 ± 0.24) and enjoyment of life (7.11 ± 1.34). Pearson's correlation analysis revealed a positive correlation between patient emotions and CRF BFI score (*r* = 0.443, *P* = 0.001), but no correlations between general activities, walking ability, work activities, relationships with others, and enjoyment of life with CRF BFI score (*P* > 0.05), as shown in Table 3.

### 3.4. Correlation between Quality of Life and Severity of CRF

The effect of the severity of CRF on quality of life was analyzed. Among the 5 items of functional dimension, physical functioning (83.34 ± 21.12 vs. 65.23 ± 21.14 vs. 32.25 ± 17.29, *F* = 15.382, *P* < 0.001) and role emotional (72.53 ± 21.21 vs. 67.33 ± 27.56 vs. 54.37 ± 26.45, *F* = 14.483, *P* < 0.001) presented statistically significant differences among patients with mild, moderate, and severe CRF. In the 9 items of symptom dimension, statistically significant differences were observed in fatigue (49.12 ± 18.44 vs. 54.61 ± 26.64 vs. 67.51 ± 14.27, *F* = 13.581, *P* < 0.001), bodily pain (56.56 ± 25.12 vs. 76.43 ± 21.71 vs. 80.32 ± 12.39, *F* = 14.582, *P* < 0.001), and appetite reduction (57.45 ± 25.47 vs. 69.51 ± 16.21 vs. 76.23 ± 27.58, *F* = 14.592, *P* < 0.001). In the dimensions of overall health status and quality of life, the CRF BFI scores of the three groups decreased successively, and the differences were statistically significant (67.21 ± 19.45 vs. 53.43 ± 22.32 vs. 43.43 ± 12.52, *F* = 16.494, *P* < 0.001), as displayed in Table 4.

### 3.5. Correlation between Short-Term Chemotherapeutic Efficacy and CRF BFI Score in Patients with Gastric Cancer

According to short-term chemotherapeutic efficacy, the patients were divided into PR, SD, and PD groups. The changes in CRF BFI score were compared among the three groups before chemotherapy, after chemotherapy, and at 3 months of follow-up. After chemotherapy, the average CRF BFI scores of the three groups all reduced than those before chemotherapy (all *P* < 0.05). At 3 months of follow-up, a comparison of the average CRF BFI scores with those before chemotherapy revealed that the average CRF BFI scores decreased in the SD and PR groups while increasing in the PD group, with statistically significant differences (*t* = 9.394, 10.384, and 7.498, respectively, all *P* < 0.05), as shown in Table 5.

## 4. Discussion

CRF is defined as a long-term and persistent subjective sense of fatigue (including physical and mental fatigue related to cancer or cancer treatment), which is not associated with recent activity and interferes with usual functioning [24]. Among tumor patients, 70%-100% have varying degrees of fatigue symptoms [25]. Many studies have confirmed that exercise can improve physical functioning, but there is insufficient evidence that exercise can improve subjective fatigue symptoms [26]. Currently, the correlation between the severity of fatigue and subjective measures of physical activity in tumor patients has not been proved by small-sample research [27]. Although there have been reports published abroad on factors relevant to fatigue and speculations about causes for fatigue symptoms, none of them have been confirmed. Consequently, the research on CRF is still in the exploratory stage.

Research on CRF in patients with advanced gastric cancer is limited in China. In the present study, the incidence of CRF in newly treated patients with unresectable advanced gastric cancer was 66.1%. The CRF BFI score was correlated with clinicopathological features such as age, PS score, TNM stage, and degree of gastric cancer thickening. The 50-59-year-old group had a higher proportion of high CRF BFI scores compared with other age groups. The higher the PS score, the higher the CRF BFI score. Wang and Lu [28] have found that CRF is correlated with PS score but not with gender, age, or pathological classification in patients with advanced non-small-cell lung cancer (NSCLC), inconsistent with our results, which may be related to a significant reduction in physical strength caused by poor eating habits and nutrient absorption disorders in patients with advanced gastric cancer.

Our results showed that CRF had a larger effect on work activities and enjoyment of life, while a smaller effect on general activities in ADLs, and the degree of CRF was significantly correlated with role emotional, which is in line with the results of Han and Yu [29]. Therefore, helping patients with advanced gastric cancer maintain normal and stable emotions is of positive significance for alleviating CRF.

In this study, it was also found that in the functional dimension of patients with advanced gastric cancer, physical functioning and role emotional were positively correlated with the degree of CRF. The severer the CRF, the poorer the physical functioning and role emotional. In the symptom dimension, fatigue, bodily pain, and appetite reduction were more obvious in patients with severe CRF. In the dimension of overall health status, the quality of life scores decreased successively in patients with mild, moderate, and severe CRF, indicating that as the degree of CRF increases, the quality of life gradually decreases. Cheng [30] and Zhang [31] have pointed out that CRF symptoms and quality of life can be improved through drug intervention (phenylmethyl acetate, modafinil, dexamethasone, vitamin D, American ginseng, etc.) or nondrug intervention (cognitive behavior therapy, proper exercise, sleep improvement, massage, acupuncture, moxibustion, etc.).

According to the study of Li [32], CRF is a symptom frequently occurring during the entire chemotherapy period. Shen and Cao [33] studied the changes in CRF of patients with advanced gastric cancer after chemotherapy and found that regardless of the chemotherapeutic efficacy, adverse reactions of chemotherapy (nausea, vomiting, bone marrow suppression, etc.) exacerbated the degree of CRF in patients with advanced gastric cancer. In our study, the CRF BFI score increased in the PD group while being significantly reduced in the SD and PR groups 3 months after chemotherapy. As a result, controlling the adverse reactions of chemotherapy in patients with advanced gastric cancer is of great significance for alleviating CRF symptoms and improving quality of life. A randomized controlled study has shown that after 6 weeks of mindfulness-based stress reduction (MBSR), CRF and sleep are significantly improved in patients with breast cancer. MBSR was developed by Kabat-Zinn of the Massachusetts Institute of Technology in the United States. Its standard intervention is performed for 8 consecutive weeks, once per week for 2 h/time, with the first and last sessions lasting 2.25 h. In the 6th week, an additional single observation (approximately 6 h) is conducted, including body scans, mindfulness yoga, meditation, mindfulness for life, and communication and listening [34]. Nevertheless, there are currently few effective treatment strategies for CRF, and relevant research has a bright future.

Of course, this study also has some limitations. Firstly, this study is a single-center study, and the included subjects are restricted to patients in our hospital and have regional origins, which may lead to a certain bias. In addition, due to limitations in human, material, and financial resources, this study only included a few subjects, which may cause insufficient power of the test. If further research is needed, the number of clinical cases will be increased according to the requirements of evidence-based medicine, and a long-term follow-up will be conducted to provide a more reliable basis for clinical treatment.

## 5. Conclusion

In conclusion, CRF is correlated with age, T stage, and N stage in gastric cancer. The later the T and N stages, the more significant the effect on fatigue. Moreover, CRF can also affect the quality of life in gastric cancer, and the severer the CRF, the poorer the quality of life.

## Figures and Tables

**Table 1:** Comparison of general data.

Item	CRF group (*n* = 152)	Non-CRF group (*n* = 78)	*χ* ^2^/*Z*	*P*
Age (year)			41.671	<0.001
20~29	4	15		
30~39	20	24		
40~49	40	23		
50~59	50	9		
60~69	38	7		
Gender (male/female)	75/77	44/34	0.034	0.855
Educational level (*n*)			-1.490	0.136
Junior high school and below	76	29		
Secondary technical school or senior high school	44	31		
Junior college and above	32	18		
Marital status (*n*)			3.453	0.327
Unmarried	6	3		
Married	118	67		
Divorced	25	6		
Widowed	3	2		
Place of residence (*n*)			0.229	0.892
Rural area	48	27		
Town	52	25		
Urban area	52	26		
Employment (*n*)			0.333	0.846
Employed	82	39		
Unemployed	42	23		
Retired	28	16		
Per capita monthly income of households (*n*)			-0.662	0.508
<3000	49	22		
~3000	48	35		
~4000	23	13		
≥5000	31	8		
Medical payment mode (*n*)			3.291	0.349
New rural cooperative medical system	45	31		
Urban resident medical insurance	51	21		
Employee medical insurance	36	14		
Self-funded	20	12		

**Table 2:** Comparison of pathological data.

Item	CRF group (*n* = 152)	Non-CRF group (*n* = 78)	*χ* ^2^/*Z*	*P*
Degree of differentiation (*n*)			1.040	0.308
Low and moderate	73	43		
High	79	35		
T stage (*n*)			9.973	0.019
T1	13	8		
T2	43	37		
T3	83	30		
T4	13	3		
N stage (*n*)			—	<0.001^∗^
N0	0	2		
N1	34	43		
N2	116	33		
N3	2	0		
PS score (*n*)			—	<0.001^∗^
0	0	43		
1	93	30		
2	59	5		
Bormann classification			7.053	0.070
Type I	5	3		
Type II	22	18		
Type III	98	52		
Type IV	27	5		
Degree of gastric cancer thickening (mm, *x* ± *s*)	14.21 ± 3.32	12.12 ± 3.81	4.572	<0.001
Lymph node metastasis			0.091	0.999
pN0	72	37		
pN1	41	21		
pN2	23	12		
pN3a	9	5		
pN3b	7	3		
Efficacy of primary lesion (*n*)			0.616	0.735
PR	117	63		
SD	21	8		
PD	14	7		
Efficacy of lymph node			0.379	0.538
Completely disappeared	121	59		
Residual	31	12		

Notes: ^∗^Fisher's exact probability test. PR: partial remission; SD: disease stability; PD: disease progression; PS: physical state.

**Table 3:** Correlation between activities of daily living and CRF BFI score in patients with gastric cancer.

Activities of daily living	CRF BFI score	*r*	*P*
General activities	2.26 ± 0.37	0.132	0.398
Emotions	4.23 ± 1.12	0.443	0.001
Walking ability	3.45 ± 0.98	0.144	0.315
Work activities	6.23 ± 0.24	0.214	0.211
Relationships with others	3.22 ± 0.89	0.265	0.276
Enjoyment of life	7.11 ± 1.34	0.274	0.143

CRF BFI: Brief Fatigue Inventory of cancer-related fatigue.

**Table 4:** Correlation between quality of life and severity of CRF.

Item	Mild (*n* = 38)	Moderate (*n* = 60)	Severe (*n* = 54)	*F*	*P*
Functional dimension					
Physical functioning	83.34 ± 21.12	65.23 ± 21.14	32.25 ± 17.29	15.382	<0.001
Role physical	52.13 ± 17.32	50.56 ± 17.36	48.31 ± 15.03	-2.034	0.071
Role emotional	72.53 ± 21.21	67.33 ± 27.56	54.37 ± 26.45	14.483	<0.001
Cognitive functioning	23.35 ± 17.23	22.56 ± 13.67	21.46 ± 13.26	1.831	0.092
Social functioning	51.56 ± 17.64	50.23 ± 16.38	48.22 ± 12.43	2.029	0.076
Symptom dimension					
Nausea and vomiting	79.34 ± 22.23	84.53 ± 14.55	85.34 ± 24.14	-2.324	0.063
Fatigue	49.12 ± 18.44	54.61 ± 26.64	67.51 ± 14.27	13.581	<0.001
Bodily pain	56.56 ± 25.12	76.43 ± 21.71	80.32 ± 12.39	14.582	<0.001
Constipation	32.46 ± 10.42	36.22 ± 15.64	35.55 ± 18.46	2.157	0.068
Diarrhea	29.42 ± 23.23	28.32 ± 13.86	30.43 ± 21.54	1.838	0.087
Insomnia	37.64 ± 28.53	39.56 ± 18.23	38.41 ± 26.42	1.780	0.096
Anxiety	45.34 ± 23.61	47.77 ± 27.31	44.42 ± 19.14	2.094	0.073
Appetite reduction	57.45 ± 25.47	69.51 ± 16.21	76.23 ± 27.58	14.592	<0.001
Financial difficulty	50.46 ± 12.62	52.71 ± 21.11	50.76 ± 20.23	1.806	0.092
Overall health status and quality of life	67.21 ± 19.45	53.43 ± 22.32	43.43 ± 12.52	16.494	<0.001

**Table 5:** Correlation between short-term chemotherapeutic efficacy and CRF BFI score in patients with gastric cancer.

	PR (*n* = 180)	SD (*n* = 29)	PD (*n* = 21)
CRF BFI score			
Before chemotherapy	5.13 ± 2.07	4.09 ± 1.36	5.57 ± 2.85
After chemotherapy	6.52 ± 1.46	5.62 ± 2.74	7.64 ± 1.91
*t*/*P*^a^	6.469/<0.001	8.482/<0.001	7.496/<0.001
3-month follow-up	3.76 ± 1.69	3.32 ± 1.23	6.61 ± 1.73
*t*/*P*^b^	9.394/<0.001	10.384/<0.001	7.498/<0.001

Notes: ^a^Comparison before and after chemotherapy. ^b^Comparison before chemotherapy and at 3 months of follow-up. PR: partial remission; SD: disease stability; PD: disease progression; CRF BFI: Brief Fatigue Inventory of cancer-related fatigue.

## Data Availability

All data generated or analyzed during this study are included in this published article.
